# Two-stage hybrid surgical repair for aortic arch pathology with a shaggy aorta: a case report

**DOI:** 10.1186/s13019-024-02841-5

**Published:** 2024-06-18

**Authors:** Atsushi Morishita, Seiichiro Katahira, Takeshi Hoshino, Kazuhiko Hanzawa, Hideyuki Tomioka

**Affiliations:** 1 Department of Cardiovascular Surgery, Numata Neurosurgery Heart-Disease, 8 Sakae-cho, HospitalNumata, 378-0014 Japan; 2 Division of Health Administration, Hamakawasaki Operation Center, Toshiba Human Asset Service Corporation, Kawasaki, Japan; 3 Department of Anesthesiology, Minami Machida Hospital, Machida, Japan; 4 Department of Advanced Treatment and Prevention for Vascular Disease and Embolism, Niigata University Graduate School of Medical and Dental Sciences, Niigata, Japan; 5 Department of Cardiovascular Surgery, Saitama Easten Cardiovascular Hospital, Koshigaya, Japan

**Keywords:** Shaggy aorta, Total arch replacement with conventional elephant trunk, Secondary thoracic endovascular aortic repair

## Abstract

**Background:**

The surgical treatment strategy for aortic arch pathology with a shaggy aorta must be determined on a case-by-case basis because of the risk of catastrophic complications, such as brain infarction and spinal cord injury.

**Case presentation:**

This report describes the surgical case of two saccular aneurysms of the arch and abdominal aorta associated with a shaggy aorta in a 63-year-old man who underwent total arch replacement and secondary thoracic endovascular aortic repair. Considering the risk of embolization during endovascular therapy, graft replacement for the abdominal aortic aneurysm was initially performed. On postoperative day 28, total arch replacement with the conventional elephant trunk was performed using the functional brain isolation technique, which involves manipulating places far from the atherosclerotic burden, such as arterial inflow for cardiopulmonary bypass and unclamping of neck vessels. On postoperative day 7 after total arch replacement, thoracic endovascular aortic repair was performed across the conventional elephant trunk in the nondiseased descending aorta. No postoperative complications, such as cerebrovascular failure, paraplegia, or embolization to abdominal viscera or lower extremities, occurred. The patient remained asymptomatic.

**Conclusions:**

The present case suggests that total arch replacement with the conventional elephant trunk and secondary thoracic endovascular aortic repair may be an effective alternative for aortic arch pathology with a shaggy aorta. The strategy for surgical treatment in patients with aortic arch pathologies with a shaggy aorta must be judged on a case-by-case basis, considering patient characteristics, comorbidities, and preoperative evaluation using transesophageal echocardiography and computed tomography angiography, to eliminate potential determinants of intraoperative stroke.

## Background

A shaggy aorta, which is reported to be a risk factor for stroke, is considered a relative contraindication for thoracic endovascular aortic repair (TEVAR). Stroke following TEVAR remains a devastating concern, with a high incidence of up to 8.2% [1]. The underlying cause of stroke is likely to be thrombotic and atherosclerotic embolization, which is related to the manipulation of catheters and stiff guidewires and the delivery and deployment of the stent grafts. Therefore, open surgery can be primarily applied, particularly for aortic arch pathologies with a shaggy aorta, despite the widespread application of endovascular technology. Herein, we describe the case of a patient with an aortic arch aneurysm associated with a shaggy aorta who underwent total arch replacement (TAR) and secondary TEVAR, characterized by a functional brain isolation technique, an arch-first approach with retrograde brain perfusion as a cerebral adjunct, and precise secondary implantation of a stent graft into the conventional elephant trunk (CET).

## Case presentation

**Fig. 1:**
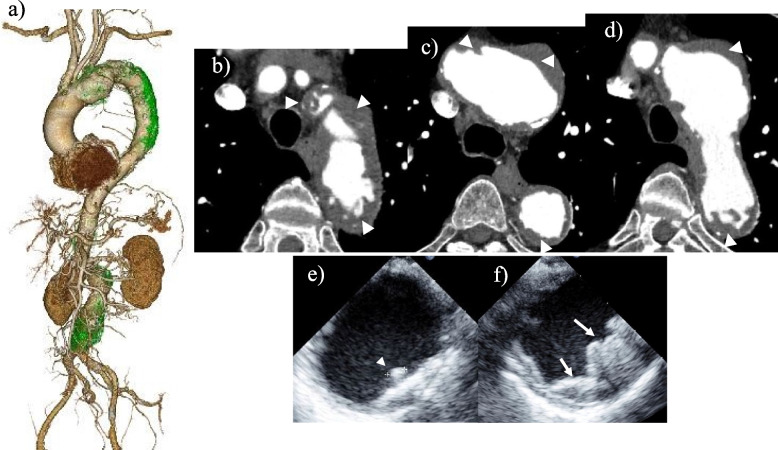
Preoperative transesophageal echocardiography and computed tomography angiography Three-dimensional reconstruction of preoperative computed tomography angiography demonstrates the ulcerated atherosclerotic burden (green color) which was recognized in the orifices of neck vessels and thoracic descending aorta adjoining the arch aneurysm (**a**). Preoperative computed tomography angiography demonstrates ulcerated or pedunculated atheroma (white arrowheads) > 10 mm into aortic lumen (**b**, **c**, **d**). Preoperative transesophageal echocardiography demonstrates (**e**) a mobile atheroma (white arrowheads) and (**f**) an atheroma protruding > 10 mm into aortic lumen (white arrows)

**Fig. 2:**
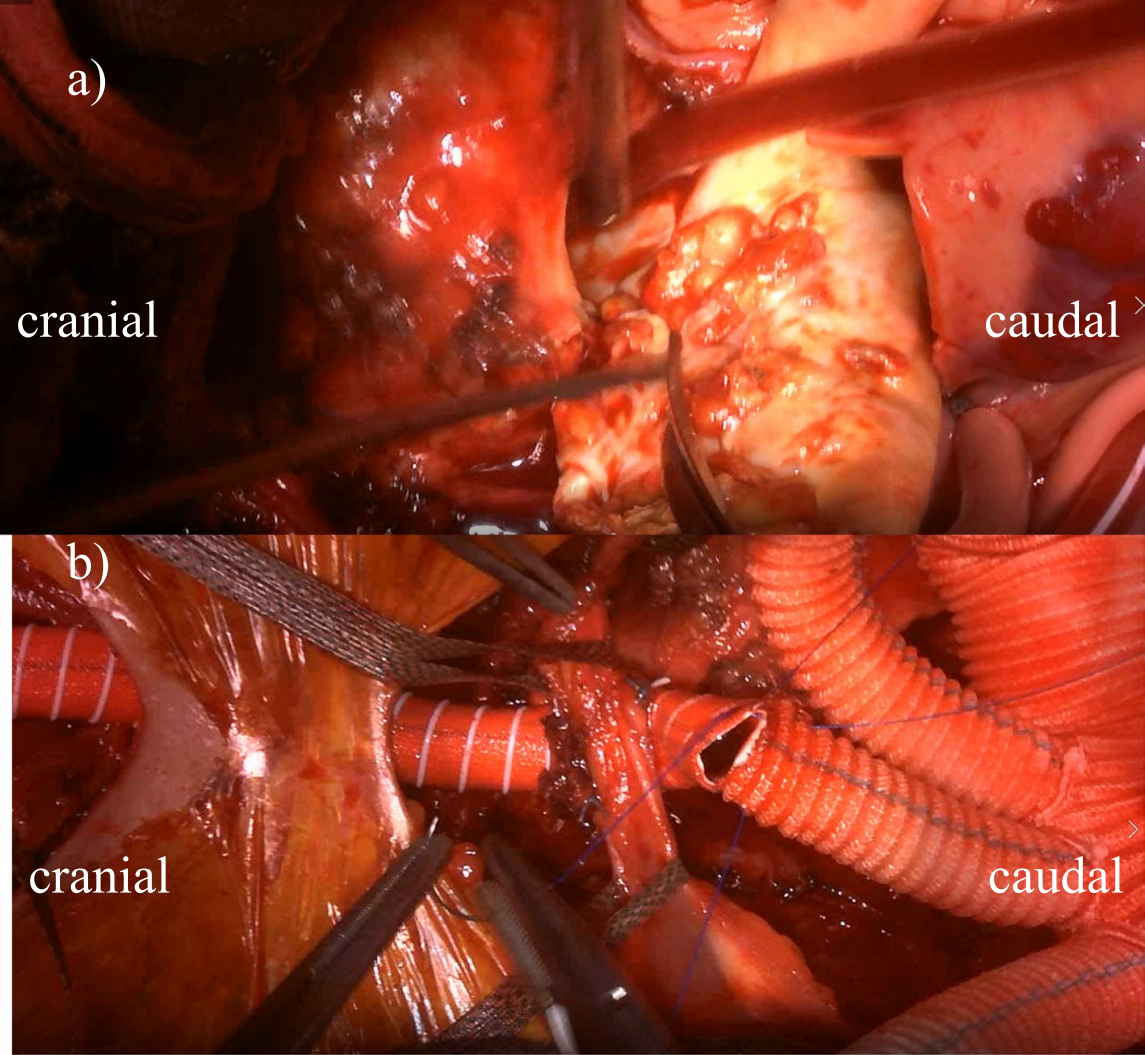
Intraoperative photographs. Intraoperative photographs demonstrates (**a**) severe atheroma around neck vessels, and (**b**) graft conduit anastomosed to the left CCA reconstructed with the side branch of the four-branch Dacron graft. CCA, common carotid artery

A 63-year-old man with left-sided hemiparesis was admitted to our hospital. The patient had been treated for hypertension and diabetes, and had no history of stroke. The patient’s height, weight, and body surface area were 178 cm, 70 kg, and 1.87 m^2^, respectively. Computed tomography angiography (CTA) revealed two saccular aneurysms of the arch and abdominal aorta with maximum diameters of 60 mm and 55 mm, respectively (Fig. 1a). An ulcerated atherosclerotic burden was observed in the orifices of the neck vessels and thoracic descending aorta adjoining the arch aneurysm (Fig. 1b, c, d). Transesophageal echocardiography (TEE) revealed heavy atheromas protruding > 10 mm into the aortic lumen with mobile components (Fig. 1e, f). Considering the risk of embolization during endovascular therapy, the patient was deemed suitable for staged traditional graft replacement. Perfusing from the femoral artery was considered essential to ensure a uniform body cooling until deep hypothermic circulatory arrest in TAR was achieved. This approach helps to prevent the embolization of the debris from the abdominal aortic aneurysm (AAA) to the abdominal viscera. After obtaining written informed consent, graft replacement for the AAA using a bifurcated woven graft was performed via median laparotomy 2 months after stroke. On postoperative day 28, TAR with CET was performed. Conventional TAR was avoided due to concerns that severe atheroma at the deep anastomotic site might prolong the the duration of deep hypothermic circulatory arrest. Moreover, Preventing stroke was deemed challenging with ascending aortic cannulation, even if the cannula tip was directed toward the aortic valve because an ulcerated atherosclerotic lesion was observed in the neck vessel orifice. The bilateral axillary arteries (AxAs) and left common carotid artery (CCA) were prepared to be individuallly constructed on an end-to-side anastomosis with an 8-mm ringed polytetrafluoroethylene graft under a simple clamp. The CCA was dissected at the proximal side of the bifurcation of the internal and external carotid artery through an additional lateral cervicotomy. The 14Fr SP stud catheter (Fuji Systems Corporation, Tokyo, Japan) was indwelled through the artificial conduit anastomosed to the left CCA, and an 18Fr FEM II cannula (Edwards Lifesciences, Irvine, Calif., USA) was inserted into the right femoral artery (FA). Oxygenated blood from cardiopulmonary bypass (CPB) was drawn from the right AxA, left CCA, left AxA, and right FA with perfusion flow rates of 1.6 L/min, 0.1 L/min, 1.2 L/min, and 1.5 L/min, respectively. Perfusion to the left CCA via the graft remained unchanged, regardless of clamping or declamping of the proximal side of the anastomotic site with a conduit, resulting in functional total occlusion in the native left CCA. The tip of the catheter that was cannulated into the left CCA was maintained at 60 mmHg. During the cooling of the whole body such that the esophageal temperature was below 20 ℃, much attention was paid to not touching the affected aorta under monitoring by near-infrared spectroscopy (NIRO 200NX, Hamamatsu Photonics K.K., Shizuoka, Japan). The orifice of the left subclavian artery was double-ligated under deep hypothermic circulatory arrest with retrograde cerebral perfusion. The cardioplegic arrest was induced via retrograde cardioplegic administration. The retrograde cerebral perfusion flow was maintained at 100 to 150 mL/min while increasing the superior venous pressure to 15 mmHg and checking the backflow from neck vessels. Subsequently, the innominate artery and graft conduit anastomosed to the left CCA were reconstructed with the side branches of the four-branch Dacron graft (J-graft 4 branched, Japan Lifeline, Tokyo, Japan) using an arch-first approach (Fig. 2). Cerebral perfusion was switched from retrograde to antegrade using a four-branch Dacron graft, and antegrade cerebral perfusion was maintained at 700 mL/min. A distal anastomosis was created behind the left CCA, in which an invaginated tube graft 5 cm in length was inserted while reinforcing the outside of the aorta by Teflon felt strip without disrupting the protruding thick atheroma. The debris was fallen into the aorta, and the air was evacuated by perfusion from the right FA. The tube graft invaginating the distal anastomotic side was retrieved and anasotomosed with the main graft using a stepwise technique, resulting in a CET that floats within the aneurysm. After the reconstruction between the main graft and graft conduit anastomosed to the left AxA through the thorax via a second intercostal space, resumption of the whole body was performed. Finally, proximal anastomosis was performed with a reinforced transected ascending aorta above the sinotubular junction. The TAR was completed. The operation time, CPB, aortic cross-clamp, retrograde cerebral perfusion, antegrade cerebral perfusion, and circulatory arrest of the lower body were 490, 225, 108, 29, 41, and 80 min, respectively.

**Fig. 3:**
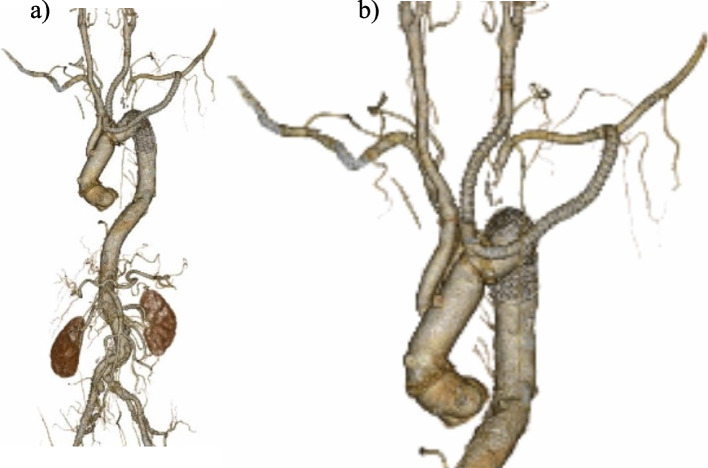
Postoperative computed tomography angiography. Three-dimensional reconstruction of postoperative computed tomography angiography demonstrates (**a**) complete exclusion of the aortic arch aneurysm after total arch replacement with the conventional elephant trunk and secondary thoracic endovascular aortic repair, and (**b**) the details of arch vessel reconstruction

TEVAR was scheduled on postoperative day 7 after TAR. A 31×150-mm conformable GORE TAG stent graft (W.L.Gore and Associates, Inc.,Flagstaff, AZ, USA) was advanced in a retrograde fashion across the CET through the right FA and subsequently deployed distal to the side branch of a four-branch Dacron graft, which is supposed to perfuse to the left arm. Touch-up via inflation of a balloon (tri-lobe balloon catheter, W.L. Gore & Associates, Inc.) was performed only in the range where the stent graft and elephant trunk overlapped. The postoperative course was uneventful, without cerebrovascular failure or paraplegia. Embolization to the abdominal viscera and lower extremities was not observed. Postoperative CTA revealed the complete exclusion of the aortic arch aneurysm (Fig. 3). The patient was discharged 38 days after TEVAR. He was followed up once a month after discharge from the hospital and remained asymptomatic.

## Discussion and conclusions

A shaggy aorta represent a category of critical importance that shows protruding eccentric plaques or thrombi on contrast-enhanced CTA finding and vulnerable aortic plaques and injuries on nonobstructive angioscopic and pathological findings. In particular, it is crucial to assess the perioperative atheroembolic risk caused by a shaggy aorta preoperatively. In view of the preoperative risk stratification, the severity of aortic arch atheroma was categorized according to the preoperative CT and TEE grades described by Gutsche et al. [1] and Katz et al. [2], respectively. The present patient was considered unfit for a total debranching TEVAR because he was diagnosed with severe grade V arch atheroma, which extends to the orifice of the innominate artery.

TAR-reinforcing brain protection is considered an effective treatment of choice for aortic arch pathologies associated with severe atheroma [3]. Furthermore, we observed strict adherence without touching the aorta until a deep hypothermic circulatory arrest was achieved. Reconstruction of the left CCA and left AxA at other incisions is a useful technique because it minimizes manipulation around the orifices of the neck vessels affected by severe atheromatous changes. Cerebrovascular events in TAR may occasionally occur because of debris embolization, which includes the non-physiological bloodstream of the extracorporal circulation, direct manipulation of the aorta and cervical vessels for cannulation or clamping, and incomplete brain protection. Furthermore, the presence of a shaggy aorta increases the risk for neurological complications [4].

Several methods for cerebral protection must be closely investigated while performing TAR. Although antegrade cerebral perfusion is widely used, it is impossible to prevent complete brain malperfusion due to incomplete intracranial vascular communication, heterogeneous perfusion of all three neck vessels, and an inadequate perfusion rate. In contrast, retrograde cerebral perfusion as a cerebral adjunct may have played an important role in preventing the dropping of plaque debris in the present case.

The brain isolation technique, which precedes and separates selective cerebral perfusion from systemic perfusion, may be a valuable adjunct for preventing cerebral embolization, as reported by Shiiya et al. [5] and Sawazaki et al. [6]; however, there is concern about the failure of brain perfusion at normal temperature, and it is not necessarily possible to clamp the neck vessels in patients with a shaggy aorta. In the present case, the functional brain isolation technique, which was composed of retrograde perfusion to the brain from neck vessels, except for left CCA, and unclamping of the orifices of neck vessels, was adopted as an effective alternative, although it was unclear whether adequate cerebral perfusion was ensured. Furthermore, it was possible that the fragile atheroma was unexpectedly stirred up to the left CCA with a turbulent jet, given that streamline analysis showed that the flow from both AxAs hit the lesser curvature of the aortic arch in the aortic arch aneurysm experimental model, as reported by Minakawa et al. [7]. This finding was sufficient to indicate the requirement for individual antegrade perfusion of the left CCA. In the present case, it was demonstrated that adding the left CCA to the bilateral AxAs as the arterial inflow for CPB could result in functional total occlusion in the native left CCA,as evidenced by individual measurements of the perfusion flow rate.

The frozen elephant trunk (FET) technique, which takes advantage of endovascular technology, has been introduced as a single-stage procedure for TAR. Even if a severe atheroma inside the aorta is visible directly under deep hypothermic circulatory arrest, a stent graft introduced by antegrade delivery, that is, the FET, may scratch and scatter a rugged shaggy arch aorta blindly and carry a high risk of debris embolization. In the present case involving CET, it is crucial to meticulously select and insert the tube graft according to the length of the aneurysm, while minimizing contact with the inner surface of the aneurysm as much as possible. The incidence of new distal stent graft-induced entries following the FET technique is reportedly higher than that following conventional TEVAR [8]. Spinal cord injury in patients undergoing FET was reported to be significantly higher than that in patients undergoing CET [9]. Spinal cord injury after TEVAR in patients with prior infrarenal AAA repair occurs significantly compared with that in patients without prior AAA surgery, as reported by Schlösser et al. [10]. In this patient with prior infrarenal AAA repair, it was crucial to pay attention to avoid intraoperative hypotension during TEVAR. There is concern that simultaneous and parallel TAR and TEVAR may cause progressive intraoperative hemodynamic instability associated with hypotension due to bleeding. Furthermore, anastomosis to the descending thoracic aorta distal to the aneurysm, requiring thoracotomy, appeared to be an invasive surgical option, particularly in the present case of comorbidities. Therefore, TAR with CET in combination with secondary TEVAR was considered to be the appropriate treatment of choice.

Patients with a shaggy aorta may potentially benefit most from the two-step approach, in which a conventional stent graft is secondarily placed in the intended place within the CET retrogradely while providing a sufficient proximal landing zone. Secondary conventional stent graft implantation supports precise deployment of the stent graft to its intended position, completely excluding the diseased aortic segment, under prudent TEE and fluoroscopic guidance. Skillful manipulation of guidewires, catheters, and devices during endovascular therapy is important to protect against embolization to the abdominal viscera and lower extremities. Neither an adjunctive technique to prevent embolization to the abdominal viscera and lower extremities nor cerebrospinal fluid drainage to reduce spinal cord injury was used in the present case. Therefore, it is crucial to consider the merits and demerits of these approaches on a patient-to-patient basis.

Additionally, TEE would surely contribute to the evaluation of severe atheroma plaques, including mobile components, as a sensitive modality and to the precise deployment of the stent graft in a nondiseased descending aorta.

In summary, our integrated treatment strategy for TAR in patients with a shaggy aorta can be described as follows: the condition of being far from the atherosclerotic burden in the arterial inflow for CPB, the functional brain isolation technique, no touching of the atherosclerotic aorta until the deep hypothermic circulatory arrest is achieved, retrograde brain perfusion to prevent the dropping of debris, conversion to antegrade brain perfusion as soon as possible after an arch-first approach, and the CET technique as a useful adjunct to TAR to provide an excellent proximal landing zone for prospective stent graft implantation in the downstream aorta. The present case suggests that TAR with CET and secondary thoracic endovascular aortic repair may be an effective alternative for aortic arch pathology with a shaggy aorta. Nevertheless, the strategy for surgical treatment in patients with aortic arch pathologies and a shaggy aorta must be judged on a case-by-case basis.

## Data Availability

No datasets were generated or analysed during the current study.

## Abbreviations

TEVAR: Thoracic endovascular aortic repair
TAR: Total arch replacement
CET: Conventional elephant trunk
CTA: Computed tomography angiography
TEE: Transesophageal echocardiography
AAA: Abdominal aortic aneurysm
AxA: Axillary artery
CCA: Common carotid artery
FA: Femoral artery
CPB: Cardiopulmonary bypass
FET: Frozen elephant trunk
